# Mechanical and Microstructural Characterization of Friction Stir Welded SiC and B_4_C Reinforced Aluminium Alloy AA6061 Metal Matrix Composites

**DOI:** 10.3390/ma14113110

**Published:** 2021-06-05

**Authors:** Kaveripakkam Suban Ashraff Ali, Vinayagam Mohanavel, Subbiah Arungalai Vendan, Manickam Ravichandran, Anshul Yadav, Marek Gucwa, Jerzy Winczek

**Affiliations:** 1 Department of Mechanical Engineering, C. Abdul Hakeem College of Engineering & Technology, Vellore 632509, India; ashraffali1986@gmail.com; 2 Centre for Materials Engineering and Regenerative Medicine, Bharath Institute of Higher Education and Research, Chennai 600073, India; mohanavel2k16@gmail.com; 3 Department of Electronics & Communication, Dayananda Sagar University, Bangalore 506114, India; arungalaisv@yahoo.co.in; 4 Department of Mechanical Engineering, K. Ramakrishnan College of Engineering, Trichy 621112, India; smravichandran@hotmail.com; 5 Membrane Science and Separation Technology Division, CSIR-Central Salt and Marine Chemicals Research Institute, Bhavnagar 364002, India; anshuly@csmcri.res.in; 6 Department of Technology and Automation, Częstochowa University of Technology, 42-201 Częstochowa, Poland; mgucwa@spaw.pcz.pl

**Keywords:** stir casting, boron carbide, silicon carbide, AA6061 aluminium alloy, tensile strength, friction stir welding

## Abstract

This study focuses on the properties and process parameters dictating behavioural aspects of friction stir welded Aluminium Alloy AA6061 metal matrix composites reinforced with varying percentages of SiC and B_4_C. The joint properties in terms of mechanical strength, microstructural integrity and quality were examined. The weld reveals grain refinement and uniform distribution of reinforced particles in the joint region leading to improved strength compared to other joints of varying base material compositions. The tensile properties of the friction stir welded Al-MMCs improved after reinforcement with SiC and B_4_C. The maximum ultimate tensile stress was around 172.8 ± 1.9 MPa for composite with 10% SiC and 3% B_4_C reinforcement. The percentage elongation decreased as the percentage of SiC decreases and B_4_C increases. The hardness of the Al-MMCs improved considerably by adding reinforcement and subsequent thermal action during the FSW process, indicating an optimal increase as it eliminates brittleness. It was seen that higher SiC content contributes to higher strength, improved wear properties and hardness. The wear rate was as high as 12 ± 0.9 g/s for 10% SiC reinforcement and 30 N load. The wear rate reduced for lower values of load and increased with B_4_C reinforcement. The microstructural examination at the joints reveals the flow of plasticized metal from advancing to the retreating side. The formation of onion rings in the weld zone was due to the cylindrical FSW rotating tool material impression during the stirring action. Alterations in chemical properties are negligible, thereby retaining the original characteristics of the materials post welding. No major cracks or pores were observed during the non-destructive testing process that established good quality of the weld. The results are indicated improvement in mechanical and microstructural properties of the weld.

## 1. Introduction

In many industrial applications, aluminium alloys are reinforced with hard ceramic particles to enhance the mechanical properties of aluminium metal matrix composites (Al-MMCs) [1,2,3]. Aluminium MMCs are lightweight material accompanied with good thermal and electrical conductivity, high stiffness, hardness, strength, melting point and wear resistance [4,5,6]. Due to their higher processing prices, Al-MMCs are deployed only for military weapons and aerospace applications. Aluminium MMCs have further found applications in automobile products, such as pistons, engine, disk brakes, cylinder liners and drum brakes [7,8]. Fusion welding of Al-MMCs creates brittle intermetallic components within the weld region’s matrix and the reinforcement particles. The induced stress in the weld reduces the joint efficiency and reveals porosity and voids at the joint [9,10].

In the friction stir welding (FSW) process, a non-consumable rotating tool having higher toughness than the base material is pitched into the faying/butt ends of the plates to be welded. They are subject to appropriate axial force developed along the line of the joint. Pin and shoulder are the two key portions of the tool. The material experiencing the rotational movement of the tool pin is unstiffened by the frictional heat generated by the tool during the spinning action. The rotating tool drives the plastically distorted material from the front towards the reverse side of the tool. Subsequent forging facilitates the achievement of the weld. Though a solid-state joining process with the absence of material melting, FSW reveals a lack of stimulated melt solidified structure in the weld zone. This addresses the technical lacuna’s encountered during the fusion welding of composites [11,12,13,14,15,16]. A few impactful literature related to the FSW of Al-MMCs has been reported [17,18,19,20,21,22]. Vijay and Murugan [22] reported that fine grain size and high tensile strength was achieved for the square pin profile of friction stir welded AA6061/TiB_2_ composite. Xu et al. [21] stated that the high tensile strength values are obtained for high tool rotating speed. Topcu et al. [20] emphasized that the hardness increases with an increase in the B_4_C reinforcement. Ali et al. [18] investigated the hardness and tensile properties of weld specimen and linked them with microstructural variation in AA6061/SiC/B_4_C composites. Palanivelet et al. [17] revealed that the nugget zone and the grain growth are affected when the time of exposure of the FSW tool is high. Wook et al. [19] achieved uniform distribution of reinforced particles in weldment due to friction of FSW tool, and SiC increases the hardness of the Al-MMCs.

Limited literature reports are available on appropriate mixing percentages of SiC and B_4_C reinforcements on Al-MMCs for which industrially acceptable mechanical property ranges and microstructural integrities for reliability are established. In particular, reports on weldability studies on these Al-MMC’s are sporadic, lacking an interdisciplinary treatment. This provides a broader scope for exploration regarding the thermal implications of FSW on microstructures, process parametric influences on weld efficiency and mechanical behavioural analysis. Hence, this work is undertaken to fabricate Al-MMC reinforced with SiC and B_4_C particles using the stir casting technique in various percentage compositions. FSW is performed on these composites by varying the process parameters to the weld’s changes in properties and behaviours. The energy generated during the FSW process in terms of heat is also estimated to examine the thermal behaviour of the weld in different regions of the joint, which governs the microstructural grain sizes and the consequential, mechanical properties.

## 2. Experimental Procedure

The experimentation is carried out in two phases: the first phase involves stir casting to fabricate Al-MMC’s with varying reinforcement composition. The second phase involves the FSW of the stir cast samples, followed by testing and characterization.

### 2.1. Fabrication of Composites (Phase-I)

A graphite crucible in electrical furnace (SwamEquip, Chennai, India) was used to prepare the Al-MMCs reinforced B_4_C and SiC. Hexachloroethane tablets (M/s Madrad Fluorine Private Ltd., Chennai, India) were used for degassing (eradicate dross) from the composites. At 650 °C, B_4_C and SiC were pre-oxidized for 2 h and transferred into the liquid matrix (AA6061) with constant stirring. To improve the metal bonding, heat treatment was given to B_4_C to form a layer on SiC. After adding B_4_C and SiC at an optimal speed (500 rpm) and time (10 min), the melt was then transferred into an iron die mould. There were no traces of casting defects. To improve the wettability of the reinforced particles, magnesium was added during the stirring and melting of the alloy.

### 2.2. Experimental Setup

**Figure 1:**
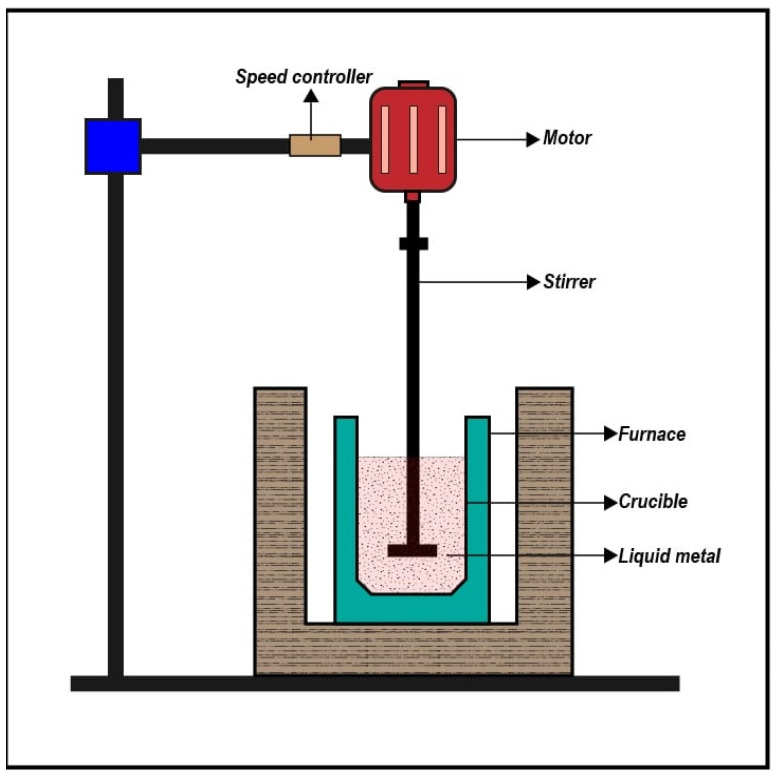
Schematic of stir casting setup.

The Al-MMCs were prepared using the stir casting unit (Figure 1). The scientico electrical furnace of dimension 600 mm × 600 mm × 600 mm of the outer shell and furnace height 0.75 m was used. The crucible (dimension 200 mm × 300 mm × 50 mm) made of Zirconia coated AISI310 stainless steel material was employed with speed variations ranging between 300 and 500 rpm. The furnace operated at 230 V, 6 kW, and the molten metal working temperature was 990 ± 0.1 °C with a maximum sustainable temperature of 1200 °C. The stir casting furnace specifications are given in Table 1.

### 2.3. Experimental Procedure for FSW of Al-MMC’s (Phase-II)

**Figure 2:**
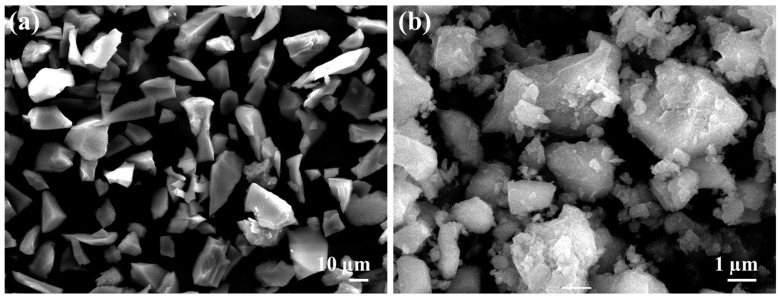
Scanning electron microscope micrograph of reinforcement particles: (**a**) SiC, (**b**) B_4_C.

To strengthen Al-MMCs, SiC (325 nm mesh size) and B_4_C (30 nm) particles were reinforced in the metal matrix (Figure 2). Hexachloroethane tablets was used to improve SiC and B4C powder’s wettability and enhance their behaviour in Al melts [23]. The composition percentages of different samples fabricated in this study are listed in Table 2.

**Figure 3:**
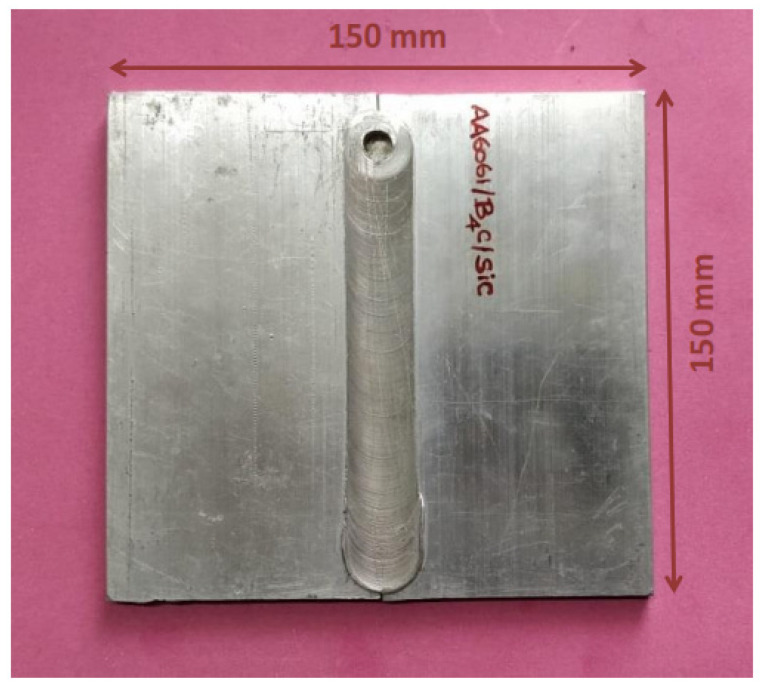
Friction stir welded specimen.

**Figure 4:**
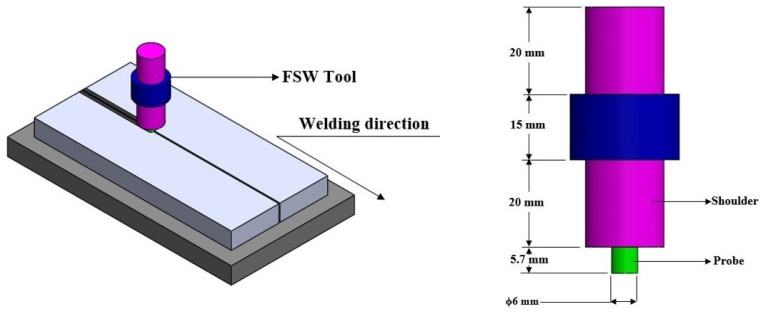
Schematic representation of FSW process and tool.

The workpieces of dimensions 150 mm × 150 mm × 6 mm were prepared in butt welding configuration (Figure 3). The FSW tool was made of tool steel (H-13) with a shoulder diameter of 25 mm. The cylindrical tool dimensions of 6 mm diameter and 5.7 mm height were considered (Figure 4). After a few trial-and-error experiments using FSW 3T 300NC, the optimal process parameters ranges were identified. The subsequent trials were based on the design of experiments (DOE) with different levels of process parameters accounting for 81 trials. However, the detailed information of the DOE based trials is not discussed here as the scope of the paper focuses on properties and behaviours of the weld. The FSW tool rotating speed, travelling speed and plunge force used for the trials were 1000 rpm, 75 mm/min and 10 KN, respectively. The welded specimens are cut along the cross-sections for tensile test specimens per ASTM (E8M) standards.

## 3. Estimation of Energy Generated in FSW for Heat Input Examinations

The amount of heat generated in FSW is the power transformed to the total amount of heat energy and expressed by Equation (1).

(1) Q=ηP

where *η*—power transformation coefficient, and *P*—power transferred.

The total amount of heat energy transferred is the sum of the translational (*Q_t_*) and rotational (*Q_r_*) heat energies as shown in Equation (2).

(2) $$Q=Q_{t}+Q_{r}$$

The total amount of mechanical power derived is dependent on the amount of torque (*M*) and angular frequency (*ω*) and is expressed by Equations (3) and (4).

dP=ωdM=ωrdF=ωrτdA

(3) P=ωM

(4) dP=ωdM=ωrdF=ωrτdA

where *r*—radial distance, *F*—force exerted by the FSW tool, *τ*—sheer contact stress in the material, and *A*—area of the weld.

By estimating the coefficients, the total heat input generated by the FSW tool is given by Equations (5) and (6):

(5) $$Q=\int_{0}^{2\pi }\int_{0}^{R}\omega r^{2}\tau d\theta dr$$

(6) $$Q=\frac{2\pi \eta }{3S}\times \mu \times F_{N}\times \omega \times R$$

Heat input in Equation (6) is the amount of energy per unit length of the weld.

## 4. Results and Discussion

### 4.1. Mechanical Testing for Strength Assessment of Welds

#### 4.1.1. Tensile Tests

**Figure 5:**
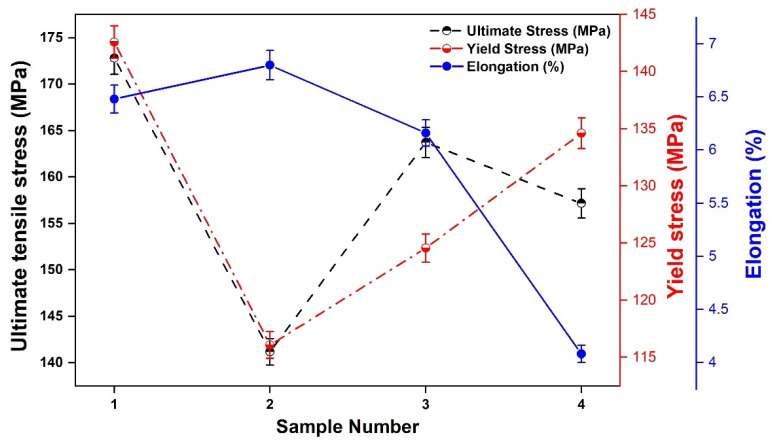
Tensile test properties measured for different sample compositions.

The tensile tests were carried out at room temperature using a 40 tonnes capacity universal testing machine (UTM) INSTRON 8801 (High Wycombe, UK). The specimens were prepared according to the ASTM (E8M) standard. It was observed that sample #1 had the lowest B_4_C content had the highest tensile strength (172 MPa) compared to the other samples (Figure 5). This is attributed to the good plasticity and density of Al [13]. With the increase in B_4_C and a decrease in SiC content in the friction stir welded Al-MMCs samples, the tensile strength decreases. This can be attributed to the even distribution of fine Al-SiC particles. Higher percentages of SiC contributed to particle agglomeration of Al-SiC during the solidification that caused the decrease in strength of composites. The maximum value of yield stress was 142.58 MPa for sample #1, within the acceptable range of most applications. The strengthening of the composites joints after the FSW process may be attributed to dislocation density of SiC and B_4_C particles, plastic deformation, and interactions between dislocations. The high yield strength of the Al-MMCs was due to the high amount of dislocations in its matrix. During the synthesis of Al-composite, additional dislocations were introduced by SiC and B_4_C particles due to the induced internal stresses. It is attributed to the different cooling rates of the reinforcement particles from the weld process temperature due to the thermal expansion coefficients mismatch. The percentage elongation drastically reduced with a decrease in SiC and an increase in B_4_C content. SiC exhibits high strength to weight ratio and high strength retention at elevated temperatures. With this, composite may also improve chemical stability and eliminate catastrophic failures when added to a metal matrix. However, it is important to account that processing of SiC at high temperature is preferable only when the reinforcement is to be processed at high temperature. There is also maximization of monolithic ceramics causing fragility in the composites. During the FSW process, the thermal expansion coefficient between reinforcement and the matrix contributes to the thermal cooling stress from processing temperature.

#### 4.1.2. Hardness Test

**Figure 6:**
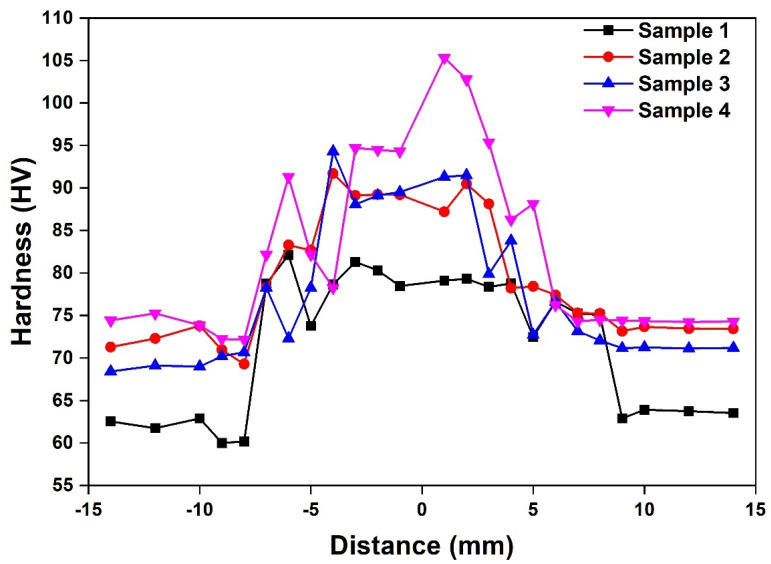
Comparison of hardness for friction stir welded Al-MMCs samples.

The interface bonding strength between the Al matrix and reinforcement particles was recorded using the microhardness tests (Figure 6). It was observed that sample #4 had higher hardness followed by samples #3, #2 and #1. This observation emphasizes that the hardness is directly proportional to the B_4_C and inversely proportional to the SiC content. Unreinforced Al alloy had an average hardness value of 23 HV, which improved considerably by adding reinforcement and subsequent thermal action during the FSW process, indicating an optimal increase as it eliminates brittleness. SiC and B_4_C particles are relatively harder and tightly bonded, constricting dislocation movements in the Al matrix, thus enhancing the hardness. SiC has lower thermal expansion and can withstand high temperature due to its high creep and oxidation resistance reflected in the mechanical properties when added as reinforcement in Al-MMCs. Hence, a higher SiC content results in a decline in hardness. The hardness observed at the weld zone was higher than other zones for all the samples. It indicates the strengthening of Al-MMCs after it was subjected to the FSW process. This is due to the friction action in FSW, which aids in the uniform distribution of the reinforcement particles in the Al matrix. B_4_C exhibits low density and high hardness, which improved the structural integrity and hardness of the Al-MMCs. It also possesses excellent stability with high thermal loading, wettability and abrasive capacity leading to prominent improvement in the mechanical properties.

#### 4.1.3. Wear Analysis

**Figure 7:**
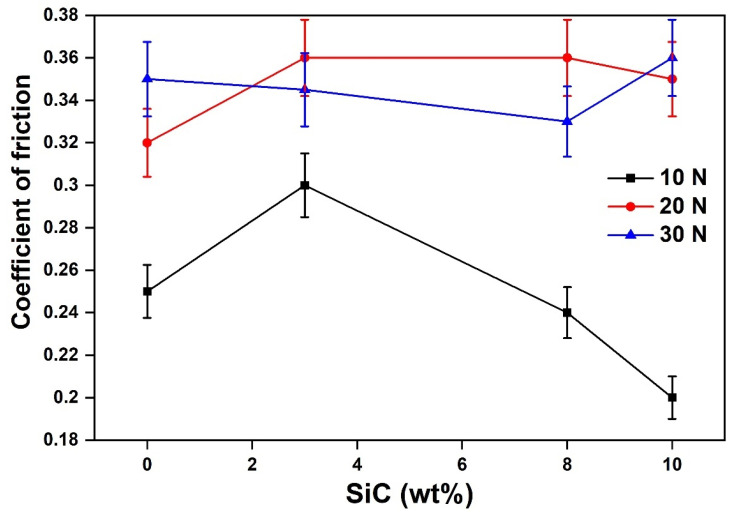
Variation of coefficient of friction of Al-MMCs with SiC content at different load and constant velocity of 1 m/s.

**Figure 8:**
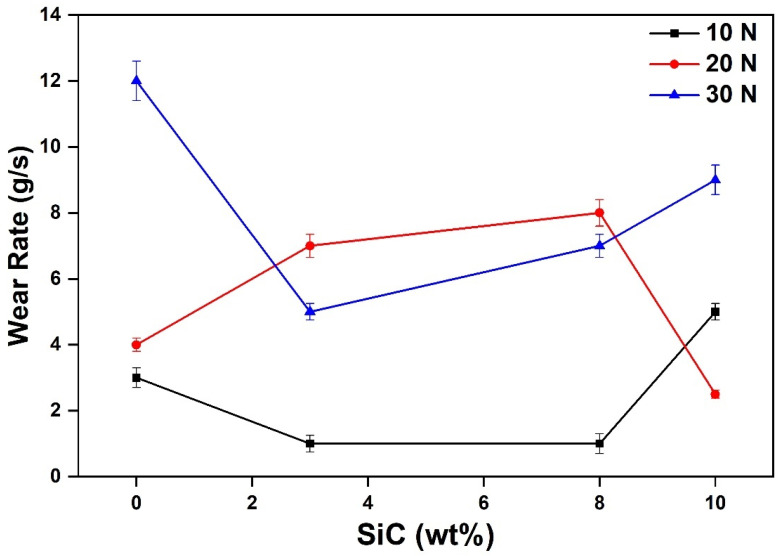
Variation of wear rate of Al-MMCs with SiC content at different load and constant velocity of 1 m/s.

Wear tests were performed on SiC and B_4_C reinforced Al-MMCs to study the wear behaviour of Al-MMCs. B_4_C particles showed an insignificant impact on the wear properties, and consequently, the study was restricted to only the SiC content. SiC exhibits excellent wear and corrosion resistance for a wide range of temperature reflected in the Al MMC’s tribological properties. For varying SiC content, the friction coefficient was recorded. The coefficient of friction was higher for samples whose SiC content ranged between 2 to 4% (Figure 7). It was also observed that the friction coefficient increased with an increase in load from 10 N to 20 N (while maintaining a constant velocity of 1 m/s). However, the friction coefficient decreased when the load was further increased to 30 N. This may be attributed to the increased brittleness of the samples [5]. The wear rate prominently increased with higher loading, and this observation was for all loading (Figure 8). The wear resistance gets transformed from abrasion to adhesion, reducing the average friction coefficient [5]. The wear rate of composites increased with the increase in load. This is due to the direct metal to metal contact, which leads to wear. The reinforcement particles reduced the plastic deformation by constricting the movement of dislocations. With the increased normal load, the reinforcement particles eroded from the surface leads to base Al resulted in a higher wear rate.

**Figure 9:**
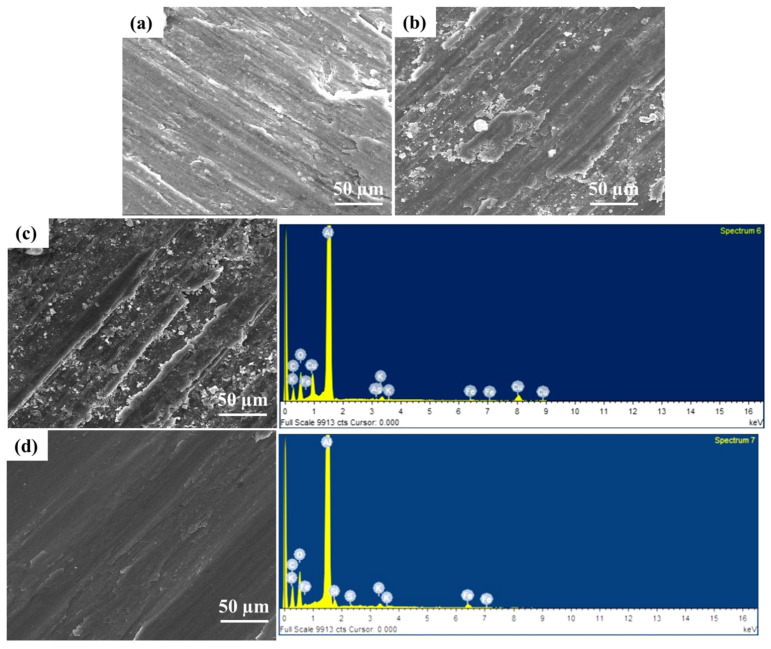
SEM/EDAX for AA 6061 composites with different reinforcement: (**a**) 10%SiC/3%B_4_C, (**b**) 8%SiC/5%B_4_C, (**c**) 5% SiC/8% B_4_C, (**d**) 3% SiC/10% B_4_C.

SEM analysis was used to investigate the material loss from the surface. The SEM micrographs of worn surface are shown in Figure 9a,b in 10 N and 20 N loads, respectively. The presence of wear debris and ploughing marks are visible, which are insignificant to cause any behavioural changes of the material. The reinforced particles agglomerated in the weld region due to the frictional heat produced during welding. The SEM micrographs of 30 N load are shown in Figure 9c,d. The corresponding EDAX analysis is also presented. The peaks of Al and reinforcement particles were observed. Small peaks of Fe were also observed, suggesting the abrasion of the steel surface by the reinforcement particles. Particles were homogeneously distributed, and no damage of worn surface was observed for higher load. In few cases, the presence of delamination with intense plastic deformation suggests adhesion wear [5]. The peaks of O in the EDAX analyses indicate the presence of oxidative driven wear. It is well known that Al readily reacts with atmospheric oxygen and forms aluminium oxide at the surface when undergone the counter steel abrasion.

### 4.2. Radiography Testing of Welds for Quality Assessment

**Figure 10:**
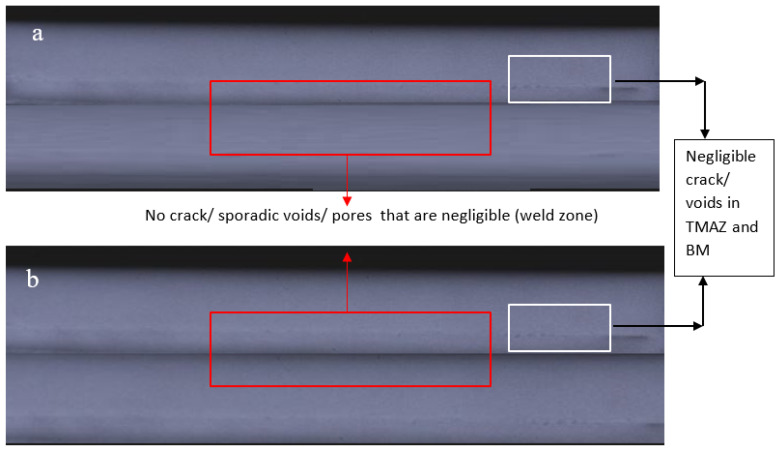
(**a**) Radiographic images of AA6061 welded composite plates reinforced with: (**a**) 5% SiC/8% B_4_C, (**b**) 3% SiC/10% B_4_C.

Al-MMCs were placed between the radiation source (Ir-192, Co-60 and Cs-137) and radioactive film. The variation in the image intensities was observed to analyse pores, cracks or discontinuities. Figure 10 shows that the friction stir welded samples (representative) were free from any defect and discontinuity. Pores or pore clusters in the weld samples may weaken the strength in the region but do not alter the weld properties. No pores or pore clusters were observed in the samples. Hence, the samples were free from defects and discontinuities.

### 4.3. Metallurgical Characterization for Assessment of Weld Microstructures

**Figure 11:**
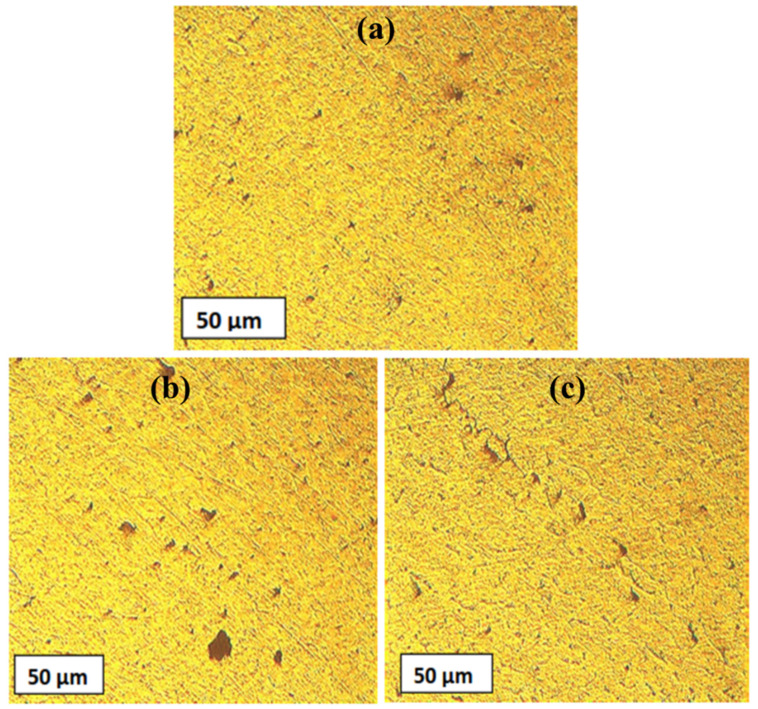
Distinct microstructures of Al-MMC: (**a**) base metal, (**b**) stir zone, (**c**) thermomechanical affected zone/stir zone.

The distinct macrostructures of different zones (base metal, stir zone, thermomechanically affected zone, heat affected zone) in the friction stir welded Al-MMCs samples are shown in Figure 11. The interfacial boundary was visible on both the advancing and the retreating side. The base material showed fine and enlarged grain particles homogeneously distributed (Figure 11a). At the extremities of the stir zone, the morphology of the microstructure significantly gets altered. (Figure 11b). In the stir zone, dynamic crystallization occurred due to the FSW process. Grain refinement occurred due to heat, mechanical deformation and stirring action of the FSW tool (Figure 11b). The boundary between the thermo-mechanical affected zone and stir zone was not visible, and the grains were distorted, aligned. A sharp transition occurred between these two regions (Figure 11c). Mechanical vibration and heat in the thermomechanical affected zone were less compared to the stir zone.

**Figure 12:**
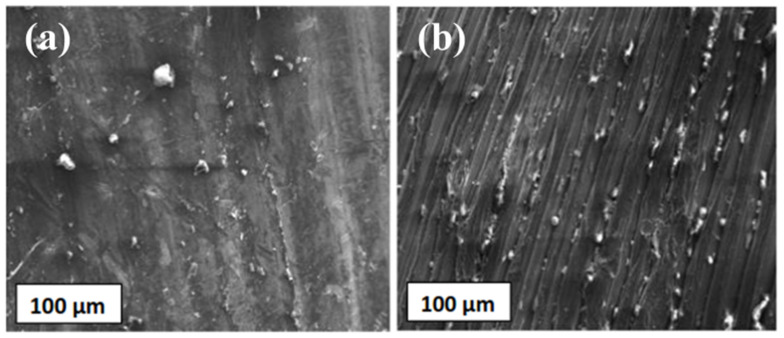
High-resolution SEM images of dynamic recrystallized zones of Al-MMC: (**a**) stir zone, (**b**) onion rings.

The microstructural examination at the joints reveals the flow of plasticized metal from advancing to the retreating side. The dynamic recrystallized zones of samples obtained from the FSW are shown in Figure 12a,b. It was observed that the reinforcement particles were distributed uniformly, and no undesired higher order reactions in the dynamic recrystallized zones were seen. It is in coherence with other reports [24,25,26]. The formation of onion rings was observed from Figure 12b due to the composite’s cylindrical FSW rotating tool material extrusion. There were no traces of common defects to FSW processes such as tunnels, wormholes and piping due to B_4_C dislocation in the Al matrix. The extensive stirring action of FSW resulted in the formation of homogenous and refined grains at the weld zone.

**Figure 13:**
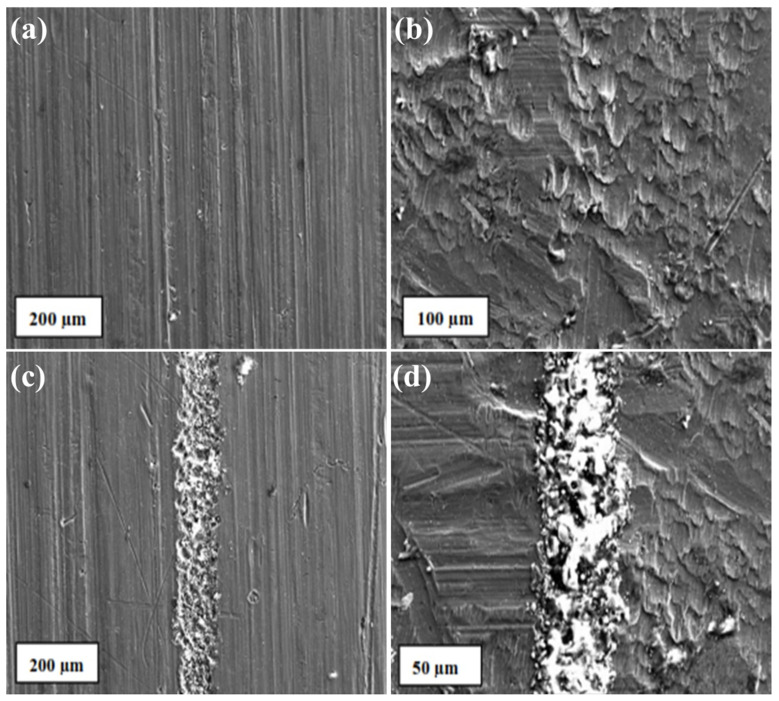
SEM images of weld zone of Al-MMC: (**a**) base material, (**b**) initial stage, (**c**) progressive stage, (**d**) advanced stage.

Figure 13 reveals the microscopic image of the weld cross-section and different zones in friction stir welded samples. Considerable change was visible in the particle size of the reinforcement phase in different regions of the joint. The reinforcement particles in the weld region revealed fragmentation in the superficial zone (average particle size reduction was about 6 times, whereas in the advancing zone average particle size reduction was about 3 times). This is because the reinforcement particles at the weld are subjected to higher heat flux compared to other regions. However, in the weld nugget region, fragmentation of the particles was significantly less (average particle size reduction was about 2 times). Distinct microstructures were observed at the nugget region of the composite. The homogenous distributions of refined grains were observed in the weld region are mainly attributed to the plastic deformation due to FSW stirring tool. The reinforcement particulate precipitates were fragmented and rearranged by the stirring action. The grains splitting and size reduction lead to the evolution of microstructure that is specific in nature. There is an irregular assortment of low/ high angle boundaries along with considerable quantum of fine equiaxed grains. The microstructure revealed directional realignment indicating close to parallel to the border between the transition zone and the stir zone. This matrix structure formation may be due to the development of deformation-induced boundaries. Besides, the traces of misorientation distribution implies a close relationship between the grain-boundary formation and texture. Henceforth, the underlying microstructural process lays the platform for continuous recrystallization. The refined equiaxed grains originate from grain boundary bulging, which is reflected in concurrent development leading to discontinuous recrystallization [27,28].

**Figure 14:**
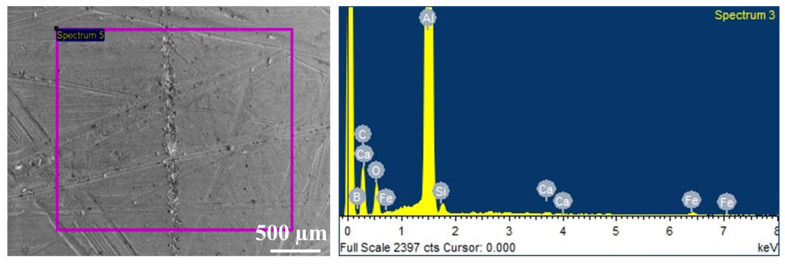
SEM with EDAX of Al-MMC in the weld nugget region.

The EDAX analysis was performed on the weld samples to identify the key elements in the joint and the adjoining regions (Figure 14). The crystalline phase particle distributions were examined by elemental mapping for Al, Si and B. It was observed that small size grains of Si and Al phases are distributed in narrow spaces available between B_4_C particle. The Al phase governs the solid solubility of various reinforcement particles. It may be noticed that Al in the SiC pellets is negligible. The uniform distribution of Al, B and Si is observed [29,30].

## 5. Conclusions

In this study, FSW was employed to join AA6061/SiC/B_4_C stir cast composites. The study aimed to identify suitable Al-MMCs with high strength, lightweight and anti-corrosion properties and explore their weldability characteristics and efficiency using FSW. The process parameters of FSW influencing the properties of the weld along with the causes attributed by the reinforcement components were investigated. The conclusions derived from this study are presented as follows:

- The tensile properties of the friction stir welded Al-MMCs improved after reinforcement with SiC and B_4_C. The strengthening of the composite joints after the FSW process may be attributed to the dislocation density of SiC and B_4_C particles, plastic deformation during joining and the interactions between dislocations. The maximum ultimate tensile stress was found to be around 174 MPa for sample #1 (10% SiC and 3% B_4_C). The percentage elongation decreased as the percentage of SiC decreases and B_4_C increases.
- The hardness of the Al-MMCs improved considerably by adding reinforcement and subsequent thermal action during the FSW process, indicating an optimal increase as it eliminates brittleness. This is due to hard and tightly bonded SiC and B_4_C particles, which constricts dislocation movements in the Al matrix.
- It was seen that wear resistance improved after the reinforcement of Al-MMCs. This is because the reinforcement particles reduced the plastic deformation by constricting the movement of dislocations. The wear rate of composites increased with the increase in load. This is due to the direct metal to metal contact, which leads to wear.
- The microstructural examination at the joints reveals the flow of plasticized metal from advancing to the retreating side. The formation of onion rings in the weld zone was due to the cylindrical FSW rotating tool material impression during the stirring action.

FSW is the feasible and appropriate process for welding Al-MMCs. The results promise to address the requirements of the aerospace and automobile industries. Due to the good weld efficiency, defence sectors like missile launchers and naval sectors with cruise components may adapt these materials along similar lines. The automobile and aerospace industries are the major beneficiaries; nevertheless, their applicability may be extrapolated to marine and defence structures.

## Figures and Tables

**Table 1:** Process parameters for stir casting.

S. No.	Parameters	Value
1	Spindle speed	300–500 rpm
2	Stirring time	10 min
3	Temperature of melt	990 °C
4	Preheated temperature of SiC and B4C particles	650 °C
5	Powder feed rate	0.75–1.0 g/s

**Table 2:** Composition of various samples in wt.%.

Sample No.	Al %	SiC %	B4C %	Mg %
1	85	10	3	2
2	85	8	5	2
3	85	5	8	2
4	85	3	10	2

## Data Availability

The authors confirm that the data supporting the findings of this study are available within the article.
